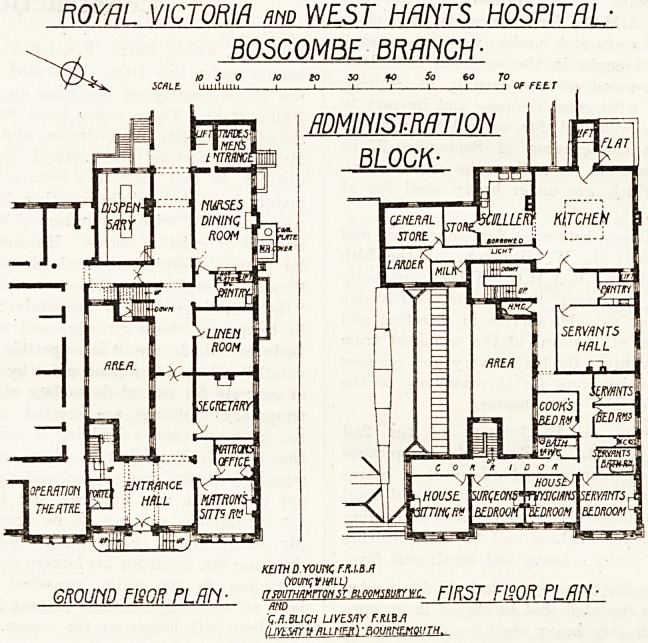# Royal Victoria and West Hants Hospital

**Published:** 1915-11-20

**Authors:** 


### HOSPITAL ARCHITECTURE AND CONSTRUCTION.

### Royal Victoria and West Hants Hospital.

BOSCOMBE BRANCH.

We illustrate to-day the ground- and first-floor plans showing the completion of the administration block, which was planned some years ago in connection with the out-patient department, the latter portion only then being carried out. Although joining on to the out-patients' department, due consideration has been given to the question of light and air by forming a large area from which the lighting of the corridors and staircases is obtained, and also additional light to the entrance hall.

It will be noticed on reference to the first-floor plan that the out-patients' block is not carried up beyond the ground-floor storey, thus giving a much larger open space and a freer circulation of air at this level.

The ground floor contains a large main entrance hall with porter's box, matron's sitting-room and an office adjoining, secretary's office, linen-room, nurses' dining-room and pantry, with a lift to the kitchen above and also provision for hot plates, etc. Beyond this is the tradesmen's entrance, with a food lift to the kitchen on the first floor. It may here be mentioned that the back part of the basement contains additional stores. There are two staircases; that in front gives access to the first-floor front, and that at the rear gives access to the first floor and servants' rooms on the second floor.

The first floor contains four rooms at the front which are ultimately intended for the resident staff. Only three of these rooms will be used at present, the fourth being cut off by a temporary partition and occupied by servants. A bathroom and w.c. is provided for the staff. At the back is a kitchen about 26 feet by 26 feet, well-lighted by windows and also a top light. Leading off the kitchen through a wide opening is the scullery, fitted with sinks, and opening out of this is a store-room. A passage lighted by a borrowed light leads from the kitchen to a good general store-room, larder, and milk store. These rooms are compact and well arranged. The servants' hall and pantry are well placed in relation to the kitchen. Four bedrooms for servants are provided on this floor, and also bathroom and w.c. On the second floor over the back part are ten bedrooms, a bathroom, lavatory, and w.c. for servants. The plan has been well considered and the general arrangement is good. Mr. Keith D. Young and Mr. G. A. Bligh Livesay acted as joint architects for the work.

## Figures and Tables

**Figure f1:**